# Maternal Hypertension after a Low-Birth-Weight Delivery Differs by Race/Ethnicity: Evidence from the National Health and Nutrition Examination Survey (NHANES) 1999–2006

**DOI:** 10.1371/journal.pone.0104149

**Published:** 2014-08-05

**Authors:** Jia Xu, Emma Barinas-Mitchell, Lewis H. Kuller, Ada O. Youk, Janet M. Catov

**Affiliations:** 1 Department of Epidemiology, Graduate School of Public Health, University of Pittsburgh, Pittsburgh, Pennsylvania, United States of America; 2 Department of Biostatistics, Graduate School of Public Health, University of Pittsburgh, Pittsburgh, Pennsylvania, United States of America; 3 Departments of OB/GYN and Epidemiology, University of Pittsburgh, Pittsburgh, Pennsylvania, United States of America; University of Louisville, United States of America

## Abstract

Studies have suggested an increase in maternal morbidity and mortality due to cardiovascular diseases in women with a prior low-birth-weight (LBW, <2,500 grams) delivery. This study evaluated blood pressure and hypertension in women who reported a prior preterm or small-for-gestational-age (SGA) LBW delivery in the National Health and Nutrition Examination Survey 1999–2006 (n = 6,307). This study also aimed to explore if race/ethnicity, menopause status, and years since last pregnancy modified the above associations. A total of 3,239 white, 1,350 black, and 1,718 Hispanics were assessed. Linear regression models were used to evaluate blood pressure by birth characteristics (preterm-LBW, SGA-LBW, and birthweight ≥2,500). Logistic regression models estimated the odds ratios (OR) of hypertension among women who reported a preterm-LBW or SGA-LBW delivery compared with women who reported an infant with birthweight ≥2,500 at delivery. Overall, there was a positive association between a preterm-LBW delivery and hypertension (adjusted OR = 1.39, 95% confidence interval (CI) 1.02–1.90). Prior SGA-LBW also increased the odds of hypertension, but the estimate did not reach statistical significance (adjusted OR = 1.21, 95% CI 0.76–1.92). Race/ethnicity modified the above associations. Only black women had increased risk of hypertension following SGA-LBW delivery (adjusted OR = 2.09, 95% CI 1.12–3.90). Black women were at marginally increased risk of hypertension after delivery of a preterm-LBW (adjusted OR = 1.49, 95% CI 0.93–2.38). Whites and Hispanics had increased, but not statistically significant, risk of hypertension after a preterm-LBW (whites: adjusted OR = 1.39, 95% CI 0.92–2.10; Hispanics: adjusted OR = 1.22, 95% CI 0.62–2.38). Stratified analysis indicated that the associations were stronger among women who were premenopausal and whose last pregnancy were more recent. The current study suggests that in a representative United States population, women with a history of preterm- or SGA-LBW deliveries have increased odds of hypertension and this risk appears to be higher for black women and younger women.

## Introduction

Women who have delivered a low-birth-weight (LBW) infant (birth weight less than 2,500 grams) are at increased risk for subsequent incidence and mortality from cardiovascular diseases (CVD) [1]–[4]. LBW occurred in 8.1% of the live births in the United States in 2011 [5]. About two-thirds of LBW infants are born preterm (PTB, delivery of an infant before 37 completed weeks of gestation), and the other LBW infants are considered to be small-for-gestational-age (SGA, most commonly defined as a fetal weight or birth weight below the 10^th^ percentile at a particular gestational week) [6]. PTB and SGA, the two antecedents of LBW [7], have distinct etiologies [8]–[10]. PTB complicates 6% to 12% of deliveries in developed countries [11]. PTB is often categorized based on clinical circumstances. Spontaneous PTB accounts for approximately two-thirds of all singleton PTBs. Infection and/or inflammation are well-established causal pathways for spontaneous PTB, especially early PTB [12], [13]. Medically indicated PTB, which accounts for the other one-third of PTBs, has a dominantly vascular etiology [14]. SGA, which accounts for approximately 5% to 7% of deliveries, is also primarily a vascular-related disorder [10], [15].

A recent study with the National Health and Nutrition Examination Survey (NHANES) 1999–2006 data suggested that giving birth to a SGA infant is strongly and independently associated with maternal ischemic heart disease [16]. Increasing evidence indicates that PTB or SGA delivery and later maternal CVD risk share some common features such as vascular endothelial dysfunction, metabolic syndrome, hypertension, and dyslipidemia [17], [18].

Hypertension is a major cardiovascular risk factor, contributing to approximately half of all CVD-related deaths [19]. Pregnancy has been viewed as a cardiovascular “stress test” for women [11]. A registry-based study indicated that women with a prior PTB had higher risk of hypertension after excluding preeclampsia cases [18]. Recently, a cohort study of 679 women suggested that women with a prior PTB had higher blood pressure eight years after the delivery [17]. In addition, hypertension before or during pregnancy increases the risk of preterm or SGA birth [20], [21]. Taken together, the evidence supports the existence of common predisposing risk factors for both LBW and hypertension. The pre-pregnancy subclinical and clinical vascular aberrations that contribute to LBW may persist after pregnancy and increase the mother’s CVD risk in their later life.

Previous studies relating LBW to maternal risk of elevated blood pressure later in life were conducted in predominantly white women [17], [18]. Therefore, those studies were not able to investigate the race/ethnicity-specific associations. Racial/ethnic differences in PTB, SGA, and hypertension persist despite substantial clinical and public health efforts [22], [23]. Hypertension is particularly prevalent and poorly controlled in blacks compared with whites [23], [24]. The prevalence of hypertension in Hispanic populations is similar to white populations; however, blood pressure control in Hispanics is not as successful as in whites [23]. It is also well acknowledged that black women experience much higher rates of PTB, SGA, and LBW than any other ethnic group in the United States [6]. Hispanic women have slightly higher PTB rates and similar LBW rates compared to white women [7], [25]. However, to date, the interrelationship among race/ethnicity, LBW delivery, and subsequent maternal blood pressure has not been evaluated.

This study sought to examine the independent associations between the two antecedents of LBW (PTB and SGA) and subsequent maternal blood pressure and hypertension in a representative United States population. This study also investigated whether the associations between PTB or SGA and subsequent maternal blood pressure and hypertension vary by race/ethnicity. Moreover, to explore the extent to which hypertension risks associated with pregnancy complications change over time, this study also stratified the population by menopausal status and time since last pregnancy. The hypothesis is that women with a prior PTB or SGA delivery will have increased odds of hypertension, and this will be more pronounced in black compared to white women.

## Materials and Methods

### Data source and study population

This study used data from the NHANES (http://www.cdc.gov/nchs/nhanes.htm) 1999–2006. NHANES are public use data files without identifiers, released by the National Center for Health Statistics. They are exempt from Institutional Review Board review under category 4 from 45 Code of Federal Regulations Part 46. NHANES is an ongoing survey on health and nutritional status designed to be nationally representative of the non-institutionalized, United States population [26]. Since 1999, NHANES has conducted a continuous annual survey using a stratified multi-stage probability design to obtain nationally representative samples, with an oversample of low-income individuals, individuals between 12 and 19 years of age, adults over the age of 60 years, blacks, and Mexican Americans. NHANES data are collected in two phases. First, the participants’ health history, health behaviors, and risk factors are obtained during a home interview. Participants are then invited to take part in a medical examination where they receive a detailed physical and laboratory examination.

**Figure 1:**
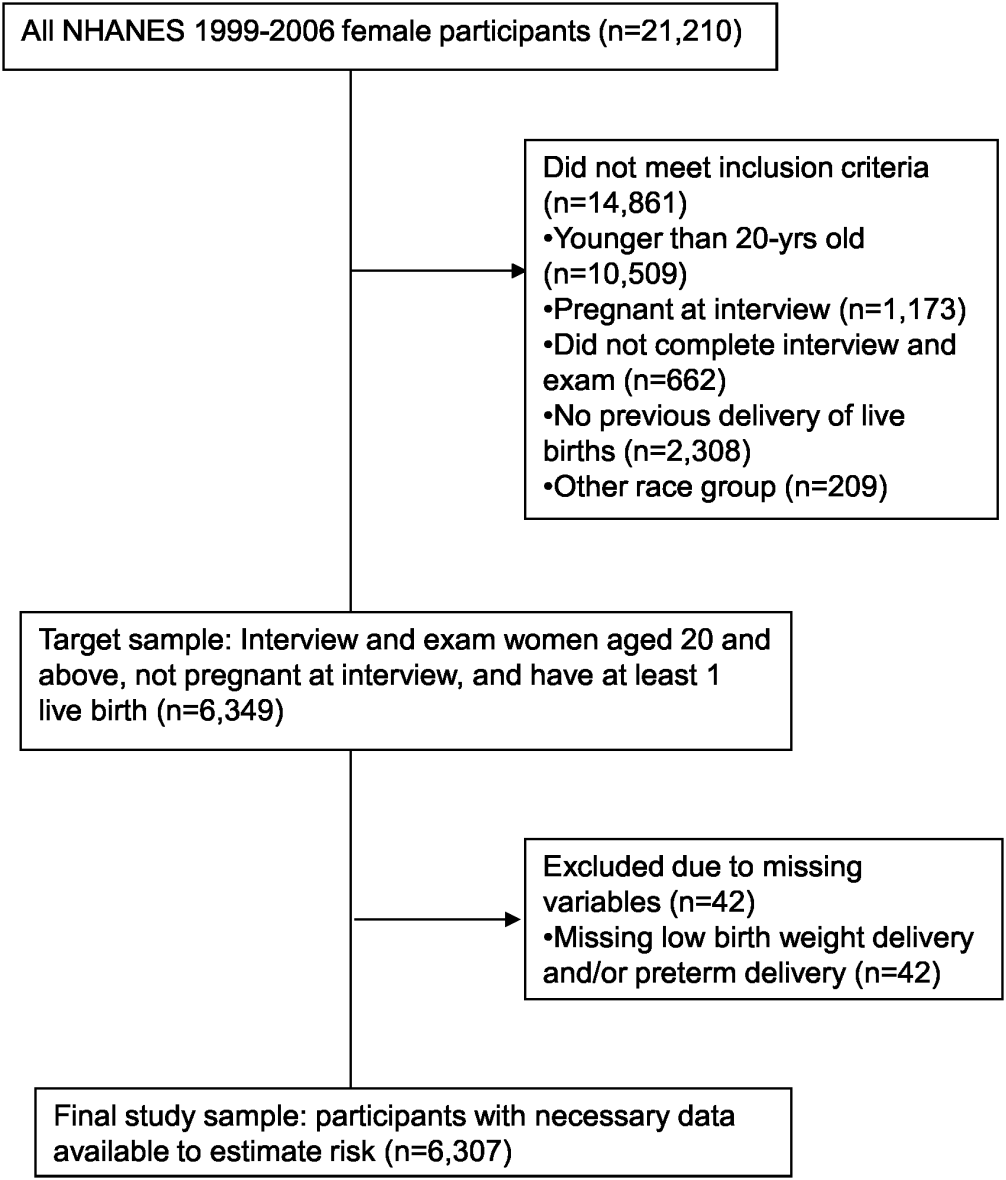
Participant flowchart. From the population of 21,210 female participants in the NHANES 1999–2006, women who were younger than 20-year old (n = 10,509), pregnant at the interview (n = 1,173), did not complete interview and exam (n = 662), did not report previous live birth delivery (n = 2,308), or were in other race group (n = 209) were excluded. This led to a target population of 6,349 women. Women who did not answer the pregnancy history questions were excluded from the target population (n = 42). Therefore, a total of 6,307 women were included in the analysis.

Of the 21,210 female participants enrolled in NHANES 1999–2006, women who were younger than 20 years of age (n = 10,509, 50%), women who were pregnant at interview (n = 1,173, 6%), women who did not complete the interview and examination (n = 662, 3%), women who did not report previous live birth delivery (n = 2,308, 11%), and women who reported race/ethnicity other than white, black or Hispanic (n = 209, 1%), were excluded in consecutive steps. Women who did not answer the pregnancy history questions were also excluded (n = 42, 0.2%) as these questions were used to construct the main exposure variable (see below). Thus, a total of 6,307 (30%) women were included in the analysis. Among these women, 3,239 (51%) women were non-Hispanic white (white), 1,350 (22%) women were non-Hispanic black (black), and 1,718 (27%) women were Mexican American and other Hispanic (Hispanic). (Figure 1).

### Study variables

#### Outcomes-blood pressure and chronic hypertension

The primary outcomes of interest were blood pressure (systolic, SBP and diastolic, DBP) and chronic hypertension. NHANES measured up to four blood pressures (BP), and the averages of these were calculated. Chronic hypertension was defined as self-report use of anti-hypertensive medication, or SBP≥140 mm Hg or DBP≥90 mm Hg at the NHANES interview.

#### Exposure-birth characteristics

The exposure variable of interest was created to represent participants’ response to two reproductive history questions. The participants were first asked “Did any child weigh less than 5.5 pounds (2,500 grams) at birth?” Women who answered yes to this LBW question were asked “How many of these babies were born preterm?” A three-level, mutually exclusive categorical variable (preterm-LBW, term-LBW, and birth weight of ≥2,500 grams) was created from these two questions to represent the birth characteristics. According to Hadlock’s study, infants born at term and with BW <2,500 grams(g) were below the 10th percentile in the United States [27]. SGA is typically defined as birth weight (BW) below the 10th percentile for gestational age. Therefore, the term-LBW group was referred to as SGA-LBW group in this study. Among 565 women in the preterm-LBW group, 106 (18.8%) had more than one preterm delivery, which were too few to be evaluated separately.

#### Covariates

Demographic, health-related, and reproductive characteristics were considered as potential confounding variables. Demographic variables included age (in years), education (less than high school graduate, high school graduate or above), and income (less than $20,000, $20,000–$45,000, more than $45,000). Health-related characteristics included insurance (having insurance/none), body mass index (BMI, the *weight* in *kilograms* divided by the square of the height in meters), waist circumference (cm), current tobacco use (yes, no), current alcohol use (yes, no), fiber in diet (gram/day), sodium intake (mg/day), physical inactivity (yes, no), family history of ischemic heart disease (yes, no), family history of diabetes (yes, no), family history of hypertension/stroke (yes, no), diabetes status (fasting blood glucose ≥126 mg/dl and/or current use of insulin or diabetes medications), and anti-hypertensive medication use (only for analysis with blood pressure as outcome). Reproductive characteristics included the number of live births, years since last pregnancy, and menopause status. Women were considered to be post- menopause if they answered “menopause” to the question “What is the reason that you have not had a period in the past 12 months?”.

### Statistical analysis

This study combined the NHANES 1999–2006 data, therefore 8-year interview/medical exam sampling weight variables were created and incorporated into the analysis to account for the NHANES sampling schemes. For continuous maternal characteristics, the race/ethnicity-specific differences in means between PTB-LBW, SGA-LBW, and BW≥2,500 g groups were evaluated using univariate weighted linear regression. For categorical variables, the differences in proportions were evaluated with Rao-Scott Chi-Square test [28]. Maternal characteristics that were significantly different among three birth characteristics groups were considered as potential confounders in the multivariable analysis. To estimate the age-adjusted prevalence of hypertension according to race/ethnicity, the population was standardized to the 2000 United States Census population with three age groups: 20–39, 40–59, and 60 and above, as recommended by National Center for Health Statistics [29].

Linear regression models were used to evaluate SBP and DBP according to LBW history, with adjustment for age at interview, race, education, insurance, cigarette smoking, waist circumference, fiber intake, sodium intake, the number of live births, years since last pregnancy, menopause status, family history of heart attack and diabetes, and anti-hypertensive medication use. Effect modification on the additive scale by race/ethnicity, menopausal status, and years since last pregnancy were assessed in the full model with potential confounders. T-tests were used to test the significance of regression coefficient of the interaction terms. If statistically significant interactions were found, stratified analyses were then performed. Sensitivity analyses were conducted among the women who were not taking anti-hypertensive medication at NHANES interview.

Logistic regression models were then developed to estimate the odds ratio (OR) of hypertension among women with a prior Preterm-LBW or SGA-LBW delivery, compared with women with a BW≥2,500 g delivery. Effect modification on the multiplicative scale by race/ethnicity, menopausal status, and years since last pregnancy was assessed using likelihood ratio tests (α = 0.05) in the full model adjusted for potential confounders. If statistically significant interactions were found, stratified analyses were then performed. In the multivariable analyses, potential confounders were the same covariates as above except anti-hypertensive medication use as this was included in the construction of the outcome.

An important unmeasured covariate in the dataset was pre-pregnancy BMI. Pre-pregnancy underweight is associated with LBW, and pre-pregnancy obesity is a risk factor for preeclampsia which is associated with maternal hypertension. In the absence of pre-pregnancy BMI data, models were additionally adjusted for BMI at age 25 as a proxy for this potential confounder. All p-values were two-sided and were considered statistically significant if less than 0.05. Statistical analyses were done using SAS 9.3 (SAS Institute Inc., Cary, NC).

## Results

Table 1 displays the race/ethnicity-specific maternal characteristics by Preterm-LBW, SGA-LBW, and BW≥2,500 g groups. The percentage of women with at least one LBW infant was highest in blacks (17.9%), followed by Hispanics (13.2%), and whites (11.8%). At the NHANES visit, black or Hispanic women were younger, had higher mean BMI and waist circumferences, had more disadvantaged socioeconomic status profiles, and were more likely to have a family history of diabetes, compared with white women. Hispanics were less likely to be insured than whites or blacks. As for clinical characteristics, compared with whites, blacks had more anti-hypertensive medication use, whereas Hispanics had less anti-hypertensive medication use.

**Table 1:** Unadjusted race/ethnicity-specific maternal characteristics of the study population: National Health and Nutrition Examination Surveys 1999–2006 (n = 6,307).

	White, non-Hispanic (n = 3,239)			Black, non-Hispanic (n = 1,350)			Hispanic (n = 1,718)		
	Preterm	SGA	BW≥2,500 g	Preterm	SGA	BW≥2,500 g	Preterm	SGA	BW≥2,500 g
	n = 257,8%	n = 126,3%	n = 2,856,89%	n = 164,12%	n = 78,5%	n = 1,108,83%	n = 144,8%	n = 83,4%	n = 1,491,88%
**Age, year**	51.3±1.0	58.8±1.6	51.7±0.4*	44.6±1.2	50.0±2.2	46.5±0.5	44.6±2.6	46.0±2.5	44.0±0.6
**Education**									
Less than highschool graduate	21%	28%	14%*	32%	48%	31%*	52%	60%	48%
**Household** **Income**									
Less than$20,000	26%	31%	18%*	37%	55%	35%	31%	59%	32%*
$20,000 to$45,000	30%	26%	32%	34%	26%	38%	45%	29%	42%
More than$45,000	44%	43%	50%	29%	19%	27%	24%	12%	26%
**Insurance**									
No insurance	15%	13%	11%	13%	13%	19%	36%	59%	35%*
**Current smoker**	33%	28%	22%*	22%	27%	21%	27%	29%	15%*
**Current alcohol** **user**	63%	64%	66%	51%	42%	45%	48%	45%	46%
**Fiber in** **diet (g/d)**	12.0±0.6	13.3±0.7	13.9±0.2*	10.7±0.5	10.9±1.1	11.6±0.3	12.3±1.1	14.3±1.0	15.7±0.6*
**Sodium** **(mg/day)**	2.7±0.1	2.7±0.1	2.9±0.1	2.7±0.1	2.4±0.2	2.9±0.1*	2.8±0.2	2.6±0.3	2.7±0.1
**Waist** **circumference (cm)**	90.4±1.0	94.1±1.8	93.9±0.4*	96.7±1.3	102.8±2.2	99.4±0.5	92.0±1.5	96.7±2.5	94.5±0.5
**BMI**	27.0±0.5	27.6±0.9	28.1±0.2	30.5±0.7	33.2±0.9	31.8±0.3	28.1±0.7	29.8±1.2	29.4±0.2
**Physical** **inactivity**	21%	29%	25%	33%	44%	31%	25%	17%	23%
**Menopause**	55%	73%	54%*	40%	55%	44%	35%	48%	36%
**Number of** **live births**									
1–2	39%	39%	58%*	43%	39%	56%*	45%	37%	50%
≥3	61%	61%	42%	57%	61%	44%	55%	63%	50%
**Years since** **last pregnancy**									
**<10 years**	24%	7%	23%*	30%	30%	30%	40%	34%	38%
**10–25 years**	34%	36%	34%	42%	28%	38%	38%	43%	40%
**>25 years**	42%	57%	43%	28%	42%	32%	22%	23%	22%
**Family history**									
Ischemic heart disease	22%	19%	19%	14%	29%	13%*	16%	3%	15%
Diabetes	47%	47%	50%	71%	63%	59%*	58%	62%	54%
HTN/stroke	35%	28%	34%	49%	59%	48%	39%	30%	32%
**Diabetes**	5%	5%	7%	11%	18%	14%	10%	12%	10%
**Anti-HTN medication**	22%	32%	24%	36%	52%	32%*	20%	10%	14%
**BMI at age 25**	22.2±0.4	21.4±0.3	22.6±0.1*	23.8±0.5	24.7±0.7	24.3±0.2	22.8±0.5	23.6±0.6	23.8±0.2

^*^p<0.05 for the test of overall differences between Preterm, SGA, and BW≥2500 g groups within each race/ethnicity group.

Abbreviation: SGA: small-for-gestational-age; BW: birthweight; BMI: body mass index; HTN: hypertension.

**Figure 2:**
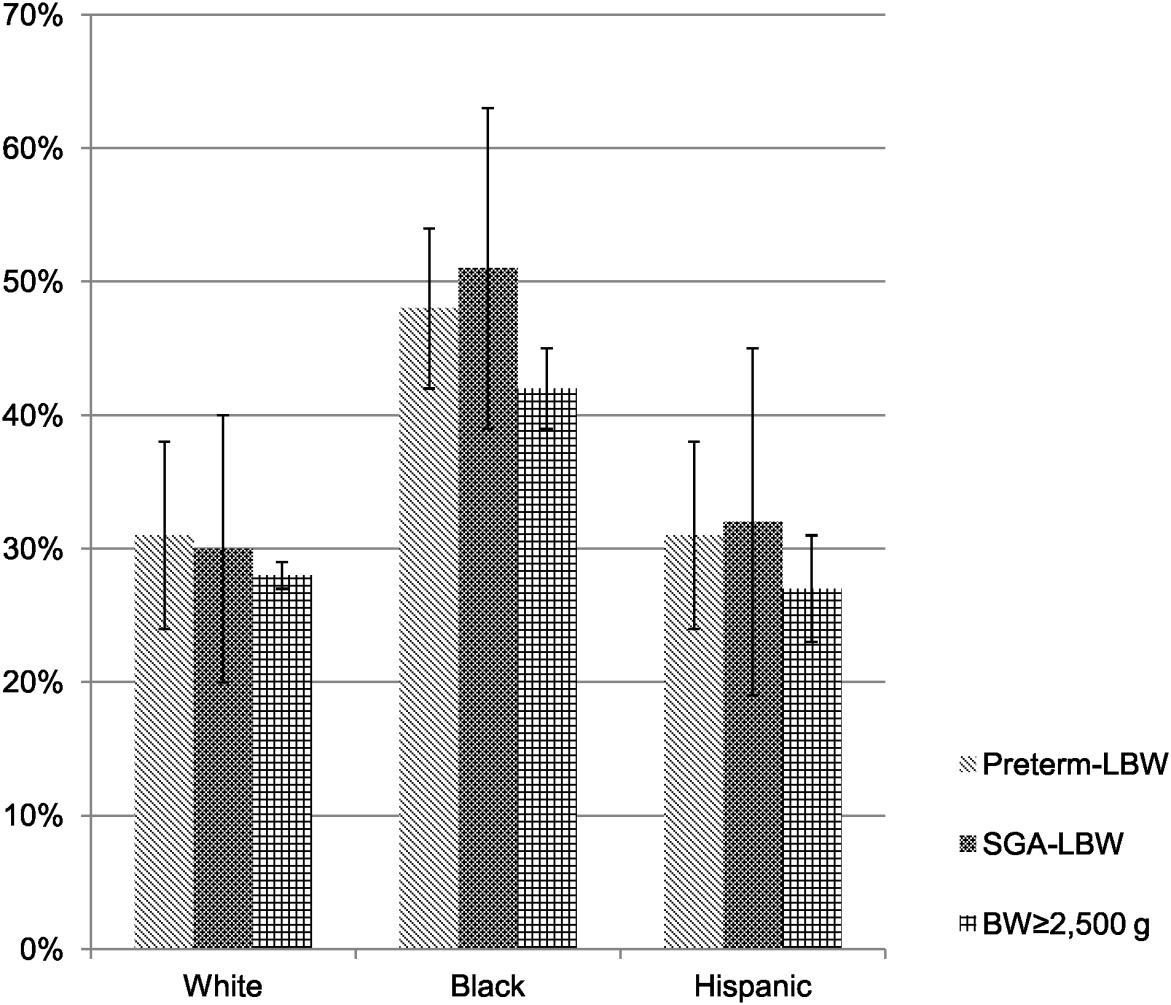
Prevalence of hypertension in white, black, and Hispanic women. Overall, black women had higher prevalence of hypertension compared to white and Hispanic women. Within each race/ethnicity group, women who had a SGA-LBW or Preterm-LBW infant had higher prevalence of HTN than the BW≥2,500 g group. It was of note that even black women with BW≥2,500 g infant delivery had higher prevalence of hypertension compared to any group of white and Hispanic women.

Overall, black women had a higher prevalence of hypertension (44%) compared to white (36%) and Hispanic (24%) women. Within each race/ethnicity group, women who had delivered a SGA-LBW or Preterm-LBW infant had higher prevalence of hypertension than the BW≥2,500 g group. Of note, black women who delivered infants with BW≥2,500 g had a higher prevalence of hypertension compared to any group of white or Hispanic women (Figure 2).

In the total study population, after adjustment for age, race, education, insurance, cigarette smoking, waist circumference, fiber intake, sodium intake, menopause status, the number of live births, family history of heart attack and diabetes, and years since last pregnancy, women with a prior Preterm-LBW delivery had higher odds of hypertension compared with women with a BW≥2,500 g delivery (OR = 1.39; 95% confidence interval [CI], 1.02–1.90, Table 2). A prior SGA-LBW was also associated increased the odds of hypertension, but the estimate did not reach statistical significance (OR, 1.21; 95% CI, 0.76–1.92). Assessment of interactions indicated that race/ethnicity, menopause status, and years since last pregnancy each significantly modified the association between pregnancy characteristics and maternal hypertension (p<0.05). Women with a prior Preterm-LBW infant had higher mean SBP compared to women with BW≥2,500 g delivery (132.6 mmHg vs. 130.3 mmHg, p = 0.01), after adjusting for the confounding variables. Women with a prior SGA-LBW delivery also had significantly higher adjusted SBP than women with a BW≥2,500 g delivery (133.3 mmHg vs. 130.3 mmHg, p = 0.04). There were no significant difference in DBP between the LBW subgroups and BW≥2,500 g delivery group. None of the interactions tested were statistically significant in the linear regression models for SBP or DBP.

**Table 2:** Adjusted blood pressure and odds ratio of hypertension: National Health and Nutrition Examination Surveys 1999–2006 (n = 6,307).

	Preterm (n = 565)	SGA (n = 287)	BW≥2,500 g (n = 5,455)
	Mean±SE	Mean±SE	Mean±SE
**SBP (mmHg)** §	132.6±0.8†	133.3±1.5†	130.3±0.6*
**DBP (mmHg)** §	69.9±0.7	68.8±1.2	69.5±0.5
	Odds ratio (95% CI)	Odds ratio (95% CI)	
**HTN** ‡	**1.39 (1.02–1.90)**	1.21 (0.76–1.92)	ref

^*^ **p<0.05 for the test of overall difference in SBP or DBP between Preterm, SGA, and BW≥2,500 g groups.**

† **p<0.05 for the test of difference in the pairwise comparison of SBP or DBP between Preterm or SGA and BW≥2,500 g.**

§ **Adjusted for age at interview, race, education, insurance, cigarette smoking, waist circumference, fiber intake, sodium intake, parity, years since last pregnancy, menopause status, family history of heart attack, family history of diabetes, and anti-hypertensive medication use.**

‡ **Adjusted for age at interview, race, education, insurance, cigarette smoking, waist circumference, fiber intake, sodium intake, parity, years since last pregnancy, menopause status, family history of heart attack, and family history of diabetes.**

**Abbreviation: SBP: systolic blood pressure; DBP: diastolic blood pressure; HTN: hypertension; SGA: small-for-gestational-age; BW: birthweight; SE: standard error; 95% CI: 95% confidence interval; ref: reference group.**

The adjusted ORs of hypertension varied by race/ethnicity groups (Table 3). In black, compared to women with BW >2500 g, the odds of hypertension were marginally higher among those with Preterm-LBW (adjusted OR, 1.49; 95% CI, 0.93–2.38; p-value = 0.10) and significantly higher among those with SGA-LBW (adjusted OR, 2.09; 95% CI, 1.12–3.90; p-value = 0.02) births. White and Hispanic women also had higher odds of hypertension after a Preterm-LBW (whites: adjusted OR, 1.39; 95% CI, 0.92–2.10; Hispanics: adjusted OR; 1.22, 95% CI, 0.62–2.38) but the estimates did not reach statistical significance. There were no observed associations between hypertension and SGA-LBW delivery among whites (adjusted OR, 1.11; 95% CI, 0.61–2.02) or Hispanics (adjusted OR, 0.86; 95% CI, 0.36–2.05). Additional adjustment for BMI at age 25 as a proxy for pre-pregnancy BMI did not change the results.

**Table 3:** Adjusted odds ratio of hypertension by race/ethnicity, menopause status, and years since last pregnancy: National Health and Nutrition Examination Surveys 1999–2006 (n = 6,307).

By race/ethnicity (n = 6,307)									
	White, non-Hispanic (n = 3,239)			Black, non-Hispanic (n = 1,350)			Hispanic (n = 1,718)		
	Preterm (n = 257)	SGA(n = 126)	BW≥2,500 g (n = 2,856)	Preterm (n = 164)	SGA (n = 78)	BW≥2,500 g (n = 1,108)	Preterm (n = 144)	SGA (n = 83)	BW≥2,500 g (n = 1,491)
	Odds ratio (95% CI)	Odds ratio (95% CI)		Odds ratio (95% CI)	Odds ratio (95% CI)		Odds ratio (95% CI)	Odds ratio(95% CI)	
**HTN** ‡	1.39 (0.92–2.10)	1.11 (0.61–2.02)	ref	1.49 (0.93–2.38)	**2.09 (1.12–3.90)**	ref	1.22 (0.62–2.38)	0.86(0.36–2.05)	ref

By menopause status (n = 6,307)									
	Premenopausal (n = 2,679)			Menopausal (n = 3,628)					
	Preterm (n = 91)	SGA (n = 25)	BW≥2,500 g (n = 1,049)	Preterm (n = 85)	SGA (n = 27)	BW≥2,500 g (n = 522)			
	Odds ratio (95% CI)	Odds ratio (95% CI)		Odds ratio (95% CI)	Odds ratio (95% CI)				
**HTN** §	2.22 (1.32–3.72)	1.32 (0.61–2.84)	ref	1.08 (0.76–1.53)	1.17 (0.66–2.05)	ref			

By years since last pregnancy (n = 6,304)									
	<10 years (n = 1,306)			10–25 years (n = 1,793)			>25 years (n = 2,935)		
	Preterm (n = 251)	SGA (n = 124)	BW≥2,500 g (n = 2,759)	Preterm (n = 157)	SGA (n = 73)	BW≥2,500 g (n = 1,060)	Preterm (n = 138)	SGA(n = 80)	BW≥2,500 g (n = 1,392)
	Odds ratio (95% CI)	Odds ratio (95% CI)		Odds ratio (95% CI)	Odds ratio (95% CI)		Odds ratio (95% CI)	Odds ratio(95% CI)	
**HTN** †	**2.96 (1.28–6.88)**	2.71 (0.90–8.14)	ref	1.36 (0.79–2.32)	1.20 (0.68–2.12)	ref	1.27 (0.86–1.87)	1.06(0.59–1.91)	ref

‡ **Adjusted for age at interview, education, insurance, cigarette smoking, waist circumference, fiber intake, sodium intake, parity, years since last pregnancy, menopause status, family history of heart attack, and family history of diabetes.**

§ **Adjusted for age at interview, race, education, insurance, cigarette smoking, waist circumference, fiber intake, sodium intake, parity, years since last pregnancy, family history of heart attack, and family history of diabetes.**

† **Adjusted for age at interview, race, education, insurance, cigarette smoking, waist circumference, fiber intake, sodium intake, parity, menopause status, family history of heart attack, and family history of diabetes.**

**Abbreviation: HTN: hypertension; SGA: small-for-gestational-age; BW: birthweight; 95% CI: 95% confidence interval; ref: reference group.**

The adjusted ORs of hypertension also varied by menopausal status and years since last pregnancy (Table 3). Associations in the pre-menopausal group were similar to what were found in the total population. In pre-menopausal group, increased odds of hypertension were observed among women after a preterm-LBW delivery (adjusted OR, 2.22; 95% CI: 1.32–3.72). A prior SGA-LBW was also associated with increased the odds of hypertension, but the estimate did not reach statistical significance (OR, 1.32; 95% CI, 0.61–2.84). No significant differences in hypertension among preterm-LBW vs. BW >2500 g or among SGA-LBW vs. BW >2500 g were observed in post-menopausal group. The study population was also stratified by years since last pregnancy (<10 years, 10–25 years, and >25 years) to understand if LBW delivery was temporally associated with maternal hypertension. Women with a shorter duration since last pregnancy (<10 years) had increased odds of hypertension after a prior Preterm-LBW (adjusted OR, 2.96; 95% CI: 1.28–6.88) or SGA-LBW (adjusted OR, 2.71; 95% CI: 0.90–8.14). No significant differences in hypertension were observed among preterm-LBW vs. BW >2500 g or among SGA-LBW vs. BW >2500 g in the 10–25 years and above 25 years groups.

## Discussion

To our knowledge, this is the first study to demonstrate race/ethnicity-specific relationships between a LBW delivery and hypertension after pregnancy. Evidence from this United States representative population suggested that race/ethnicity modified the association between a previous LBW delivery and maternal hypertension, such that risk may be higher among black women. These findings were robust to adjustments for measured confounders, such as age, education, cigarette smoking, waist circumference, sodium intake, family history of heart attack, and family history of diabetes. Secondly, this study also assessed the separate effects of PTB and SGA in order to distinguish the contributions of these two determinants of LBW. While the Preterm-LBW association with maternal hypertension was similar regardless of maternal race/ethnicity, the link with SGA-LBW appeared to be limited to black women. Thirdly, in general these associations appeared to be stronger among women with a shorter duration after last pregnancy (<10 years). The findings are consistent with the hypothesis that the link between LBW and later maternal CVD involves vascular dysfunction. This may be particularly important among black women, and these associations may be stronger in younger compared to older women.

The positive association between Preterm-LBW and subsequent maternal hypertension is consistent with previous studies [18], [30]. The underlying mechanisms linking PTB and maternal increased odds of hypertension remain unclear. One potential mechanism may be inflammation. It is known that inflammation is causally related to spontaneous PTB, especially early PTB [12]. One study has indicated that many years after delivery, women with a prior preterm versus term birth had increased C-reactive protein (CRP) levels, a marker of acute and chronic inflammation [31]. Inflammation is also potentially implicated in the development of cardiovascular diseases [32]. It seems plausible that women with a pro-inflammatory status during pregnancy delivered preterm infants, and after delivery this pro-inflammatory status may persist and relate to arterial stiffness, subclinical cardiovascular diseases, and hypertension later in life. Alternatively, preeclampsia is associated with preterm delivery and later life maternal risk of hypertension and may explain the associations we detected. NHANES did not collect information on preeclampsia, however, adjustment for preeclampsia has not eliminated the significant association between PTB and maternal hypertension detected in a previous study [18]. Because obesity is a risk factor for preeclampsia and accounts for about 20% of preeclampsia [33], BMI at age 25 was used as a proxy for pre-pregnancy BMI in the current analysis and results did not change suggesting that our findings may be independent of this potential confounder. In the race/ethnicity-specific analysis, the ORs of hypertension were similar across white, black, and Hispanic groups for women with a prior preterm-LBW delivery (ORs ranging from 1.22 to 1.49). This study detected borderline statistically significant increased odds of hypertension in white (adjusted OR, 1.39; 95% CI, 0.92–2.10) and black women (adjusted OR, 1.49; 95% CI, 0.93–2.38), but did not detect any difference in hypertension among Hispanic women. Unlike a previous study that reported a significantly higher risk of developing hypertension after a PTB in Danish women [18], the estimates in the current study did not reach statistical significance in white women. It may be due to the nature of the NHANES data collection. In the present study, PTBs delivered with BW above 2,500 g were categorized in the BW of ≥2,500 g group (reference group), which might dilute the effect. Another possible reason why the current study did not detect such effects was that this study had a smaller sample size of white women than the previous Danish study [18].

Growth restriction is positively associated with maternal cardiovascular morbidity and mortality [30]. During pregnancy, the maternal cardiovascular system undergoes hemodynamic changes to facilitate placental circulation in order to guarantee fetal oxygen and nutrition supply. Women at risk of CVD may have an impaired ability to adjust to this hemodynamic challenge and be at higher risk of placental dysfunction, the most common cause of intrauterine growth restriction and a common feature of preeclampsia. The race/ethnicity-specific analyses suggested that unlike the similar ORs of hypertension across three race/ethnicity groups found in women with a prior preterm-LBW delivery, white, black, and Hispanic women experienced different risks of hypertension after a SGA-LBW delivery. Black women with a prior SGA-LBW delivery were about twice as likely as women with a prior BW >2,500 g delivery to have hypertension (adjusted OR, 2.09; 95% CI, 1.12–3.90). No differences in hypertension were detected for white or Hispanic women after a SGA-LBW delivery. Taken together, these findings suggest that vascular dysfunction may be of particular importance in the linkage between LBW and hypertension among black women.

The associations of LBW with subsequent maternal hypertension especially among pre-menopausal women or those with a shorter duration since last pregnancy suggested that divergent pathways may link LBW and subsequent maternal risk of hypertension among young/pre-menopausal women compared to older women. Over the last two decades, there have been significant changes in the characteristics of women of reproductive age in the United States. There is an increase in women delaying childbirth into their third or fourth decade of life and an increase in the prevalence of pre-pregnancy obesity. Thus, the more recent cohort of pregnant women may be at higher risk for both LBW and hypertension than the earlier cohorts. This may explain the more pronounced association between LBW and hypertension among the younger women in the current study.

Hypertension has a well-established association with clinical cardiovascular disease. However, measures of resting BP assessed on a continuous scale can also be informative, because even within the normotensive range, increased resting BP is a major independent risk factor for future coronary heart disease. Women who delivered a Preterm-LBW or SGA-LBW infant had significantly higher SBP compared to women with a BW≥2,500 g delivery. Race/ethnicity did not significantly modify the association between LBW delivery and SBP or DBP throughout the range of blood pressure. The cut-off values used in the hypertension definition to categorize hypertension as a dichotomous variable may partly explain why race/ethnicity was a significant effect modifier in the multiplicative model but not in the additive model. Sensitivity analyses conducted among the women who were not taking anti-hypertensive medication at interview showed minimal impact on estimates. Longitudinal studies have documented that even modest decreases in the BP in the general population have the potential to substantially reduce morbidity and mortality or at least delay the onset of hypertension [10]. It has been estimated that a 2–3 mm Hg reduction of SBP in the population would result in a 6–9% overall reduction in mortality due to stroke and a 4–6% reduction in mortality due to coronary heart disease [5]. Thus, the modest differences in SBP detected in the current study among women with LBW deliveries may contribute to excess CVD.

### Strengths and Limitations

Findings of the current study must be considered in light of limitations. First, the pregnancy history data was self-reported and collected retrospectively. However, maternal recalled infant BW and gestational age are accurate and reliable when reported years after delivery. There is evidence that only 1.6% of BW would have been misclassified into low, normal or high BW and 16.5% of gestational age would have been misclassified into preterm, term or post-term based on maternal recall [34]. Second, due to data collection questions in NHANES, infants born preterm and with BW≥2,500 g were likely to be moderately preterm (delivered at 35–36 weeks) but were grouped in the reference group. Previous reports indicate increased risk of hypertension after pregnancy among women who delivered even moderately preterm infants [30], so this misclassification might bias the associations towards the null. Meanwhile, SGA infants who were born preterm were grouped into the Preterm-LBW group and these mothers may be more severely affected. In the study population, the number of participants in Preterm-LBW (n = 565) was about twice the number of participants in the SGA-LBW (n = 287), consistent with the expectation that two-thirds of LBW infants would be born preterm [6]. Therefore, the misclassification may not be excessive. Lastly, the cross-sectional nature of NHANES did not allow causal inference in the study. Because NHANES did not collect information before or during pregnancy, residual confounding might remain. BMI at age 25 was used as a proxy for pre-pregnancy BMI in the sensitivity analysis to address the limitation.

The current study had major strengths. The population-based NHANES data facilitated the examination of race/ethnicity-specific maternal hypertension after LBW delivery in a United States representative population. Additionally, the combination of 8-years of NHANES data provided a large sample size and the standardized examination in NHANES secures high precision of the outcome measurements. To overcome the potential underestimates of hypertension by using self-report alone [35], the outcome measurements in this study included both clinical examination and self-report data. Moreover, this study controlled for several potential confounding variables (such as cigarette smoking, waist circumference and diet characteristics), which were not available in many previous large registry-based studies.

### Conclusions

In summary, this study demonstrated that odds of hypertension were increased in women with a history of LBW delivery in a representative United States population. This association was particularly important for black women. Preterm or SGA delivery may identify women who could benefit from hypertension assessment and CVD prevention to reduce future morbidity and mortality, and this early marker may be of particular importance for black women.
